# Current Advances in Liver-Targeted Drug Delivery: Synthetic, Biological, and Biomimetic Platforms

**DOI:** 10.3390/ijms27177959

**Published:** 2026-09-07

**Authors:** Shynggys Sergazy, Roza B. Seidakhmetova, Yernur Zakirov, Askhat Zhilkaidarov, Damirzhan Amirbek, Zarina Shulgau, Kulzhan Berikkhanova, Alexandr Gulyaev

**Affiliations:** 1 LLP VICTUS PHARM, Astana 010000, Kazakhstan; rozabat@mail.ru (R.B.S.); victuspharm@gmail.com (D.A.); zarina.shulgau@icloud.com (Z.S.); akin@mail.ru (A.G.); 2 Private Institution “National Laboratory Astana”, Nazarbayev University, Astana 010000, Kazakhstan; yernur.zakirov@nu.edu.kz (Y.Z.); zhilkaidarov.ash@mail.ru (A.Z.); 3 University Medical Center, Nazarbayev University, Astana 010000, Kazakhstan; 4 Professor G.V. Tsoi Scientific and Educational Center of Surgery, Astana Medical University, Astana 010000, Kazakhstan

**Keywords:** liver-targeted drug delivery, nanocarriers, nanoparticles, extracellular vesicles, plant-derived nanovesicles, biomimetic drug delivery, cell-mediated delivery, hepatic stellate cells, hepatocytes, cell-specific targeting

## Abstract

Liver-targeted drug delivery offers opportunities to increase therapeutic exposure at sites of hepatic disease while limiting systemic toxicity; however, successful targeting requires more than preferential accumulation of a carrier within the liver. This narrative review critically summarizes recent advances in synthetic, biological, and biomimetic delivery platforms, including lipid, polymeric, inorganic, and protein-based nanoparticles, nucleic acid nanocarriers, extracellular vesicles, plant-derived nanovesicles, cell-mediated systems, and cell membrane-coated nanoparticles. A literature search was conducted using PubMed, Web of Science, and Embase, with emphasis on studies published during the last decade and updated through 24 August 2026. Particular attention is given to the biological determinants of hepatic biodistribution, including sinusoidal architecture, physicochemical carrier properties, protein corona formation, receptor–ligand interactions, and disease-associated alterations in the hepatic microenvironment. The review distinguishes organ-level hepatic accumulation from cell-specific uptake and productive intracellular delivery and discusses strategies directed toward hepatocytes, hepatic stellate cells, Kupffer cells and other macrophages, liver sinusoidal endothelial cells, neutrophils, and additional immune-cell populations. Major delivery platforms are critically compared with respect to the evidence supporting targeting specificity, cargo compatibility, administration route, reported safety and immunogenicity, manufacturability, analytical characterization, and translational maturity. Particular consideration is given to extracellular vesicle- and plant-derived nanovesicle-based approaches, for which biological activity, biodistribution, standardization, and scalability remain important areas of investigation. Overall, current research in liver-targeted delivery is increasingly focused on moving beyond nonspecific organ accumulation toward disease-adapted, cell-specific, and intracellularly productive delivery, although substantial biological, manufacturing, and regulatory challenges remain before many emerging approaches can achieve routine clinical translation.

## 1. Introduction

The liver is a central metabolic and immunological organ that plays a critical role in systemic homeostasis and is involved in a broad spectrum of acute and chronic diseases. Liver cirrhosis, viral hepatitis, and hepatocellular carcinoma collectively contribute substantially to global morbidity and mortality, with liver diseases accounting for more than two million deaths annually worldwide [1]. In addition to primary hepatic disorders, complications of advanced liver disease, including spontaneous bacterial peritonitis and associated systemic inflammation, further increase the clinical burden [2]. Overall, liver diseases are estimated to account for approximately 4% of all deaths worldwide, and their global burden remains substantial [3]. Despite major advances in pharmacotherapy, effective treatment remains challenging because conventional systemic administration may result in insufficient drug exposure at the required hepatic site while simultaneously increasing off-target exposure and toxicity. Importantly, the optimal therapeutic target differs according to disease pathogenesis and may involve hepatocytes, hepatic stellate cells (HSCs), Kupffer cells and recruited macrophages, liver sinusoidal endothelial cells (LSECs), or other immune-cell populations. Consequently, there is a growing need for delivery systems capable of controlling biodistribution, protecting therapeutic cargo, and improving delivery to therapeutically relevant hepatic cell populations while limiting off-target exposure.

Liver-targeted drug delivery has therefore emerged as an important strategy for increasing therapeutic exposure within the liver while reducing unnecessary systemic distribution [4,5,6]. Recent advances encompass a broad spectrum of synthetic, biological, and biomimetic platforms, including lipid and polymeric nanoparticles, extracellular vesicles (EVs), cell-mediated systems, and membrane-coated nanocarriers. However, preferential accumulation of a carrier within the liver should not be interpreted as evidence of selective delivery to a therapeutically relevant hepatic cell population. Effective targeting depends on a combination of carrier physicochemical properties, route of administration, interactions with circulating biomolecules, hepatic microanatomy, disease-dependent alterations of the liver microenvironment, and, where applicable, receptor-mediated cellular uptake. Accordingly, evaluation of liver-targeted delivery systems should distinguish organ-level accumulation from cell-specific uptake and, ultimately, productive intracellular delivery of therapeutic cargo.

**Figure 1:**
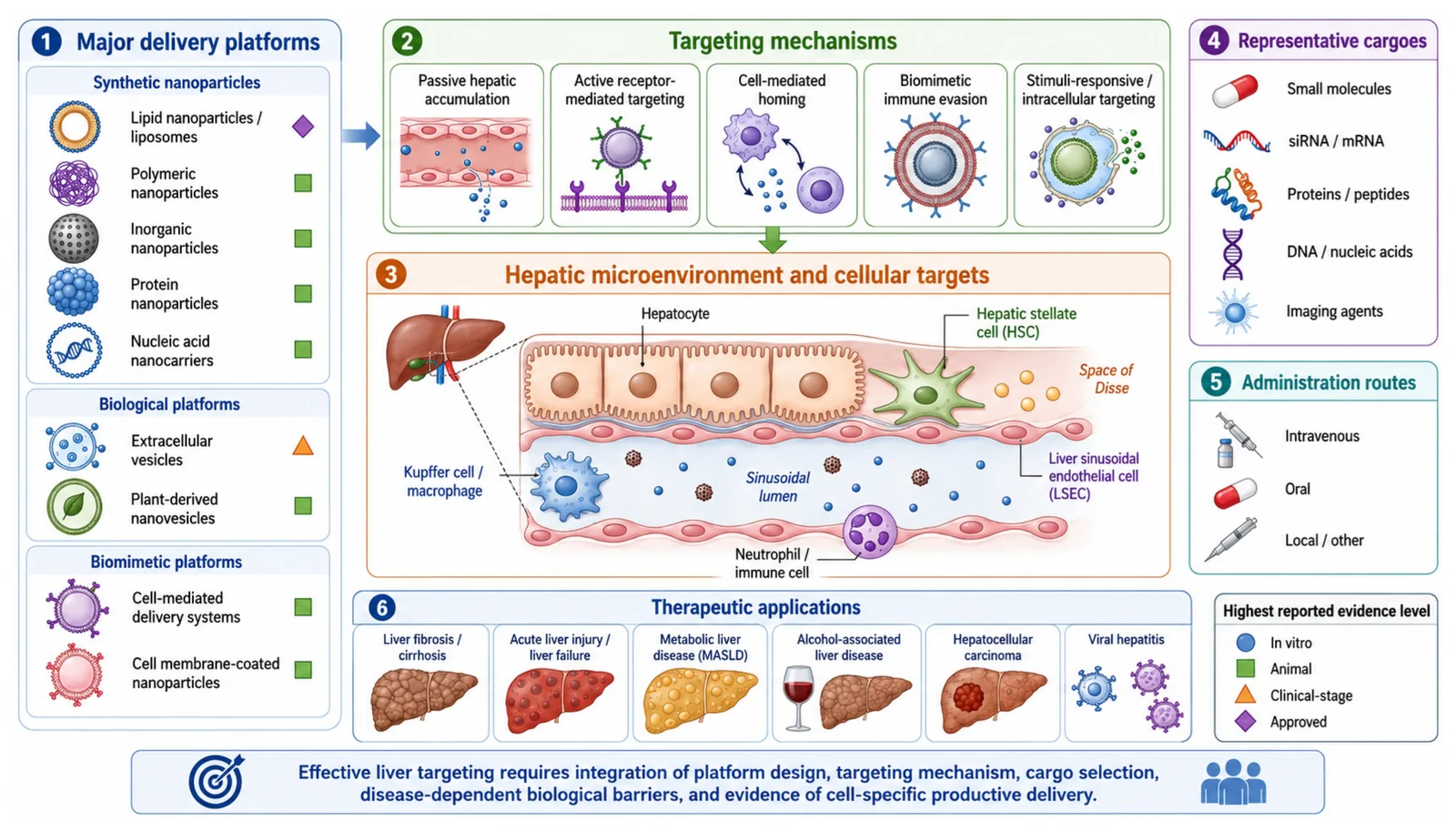
Integrative framework of liver-targeted drug delivery platforms, targeting mechanisms, and hepatic cell targets. Major synthetic, biological, and biomimetic delivery platforms are linked to passive hepatic accumulation, receptor-mediated targeting, cell-mediated homing, biomimetic immune evasion, and stimuli-responsive or intracellular targeting. These mechanisms are integrated with principal hepatic target cells, representative therapeutic cargoes, routes of administration, disease applications, and levels of evidence. Effective liver targeting requires integration of platform design, targeting mechanism, cargo selection, disease-dependent biological barriers, and evidence of cell-specific productive delivery.

This narrative review critically examines current advances in liver-targeted drug delivery across synthetic, biological, and biomimetic platforms. Rather than considering individual carrier classes in isolation, we organize the field within an integrative framework linking four interdependent components: the delivery platform, the targeting mechanism, the therapeutic cargo, and the relevant hepatic cell population. Particular attention is given to how these components interact with disease-specific biological barriers and how they influence biodistribution, cellular uptake, intracellular delivery, safety profiles, and translational potential. This framework is intended to facilitate comparison across delivery strategies and to distinguish promising experimental concepts from approaches with greater potential for clinical translation. An overview of the major platform categories, targeting mechanisms, principal hepatic target cells, therapeutic cargoes, administration routes, and levels of evidence is provided in Figure 1.

Accordingly, this review first examines the biological basis of liver-targeted delivery, including hepatic microanatomy, physiological determinants of biodistribution, and disease-associated alterations that may affect drug metabolism, vascular permeability, cellular accessibility, receptor expression, and carrier disposition. Particular attention is given to delivery barriers arising in fibrotic, inflammatory, metabolic, and malignant liver disease. The review then critically compares major synthetic, biological, and biomimetic carrier platforms, followed by an analysis of cell-specific and intracellular targeting strategies. Finally, clinical translation, manufacturing requirements, analytical characterization, safety considerations, and regulatory status are discussed to identify major opportunities and unresolved challenges for development of clinically applicable precision therapies.

## 2. Methodology

A literature search was conducted using PubMed, Web of Science, and Embase. The primary database search focused on publications from January 2016 through 24 August 2026. Search terms were used individually and in combination and included “liver-targeted drug delivery,” “liver targeting,” “nanoparticles,” “lipid nanoparticles,” “polymeric nanoparticles,” “extracellular vesicles,” “plant-derived nanovesicles,” “cell-mediated delivery systems,” “cell membrane-coated nanoparticles,” “hepatic targeting peptides,” “stimuli-responsive delivery,” “hepatocytes,” “hepatic stellate cells,” “Kupffer cells,” “liver sinusoidal endothelial cells,” “liver fibrosis,” “acute liver injury,” “metabolic liver disease,” and “hepatocellular carcinoma.” The final synthesis focused on English-language peer-reviewed publications and relevant official regulatory or clinical-trial records. Approximately 140 publications and regulatory or clinical records were assessed for relevance during preparation and subsequent revision of the review, of which 74 sources were retained and cited in the final narrative synthesis.

Because this study was designed as a narrative and integrative review rather than a systematic review, study selection was based on relevance to liver-targeted delivery, scientific quality, mechanistic contribution, and translational significance rather than on a formal systematic screening protocol. Priority was given to original research articles reporting experimental evidence of hepatic or cell-specific delivery, while high-quality reviews were used to contextualize established concepts and identify representative studies. Earlier seminal publications published before 2016 were additionally identified through backward citation screening of key reviews and were retained when necessary to describe foundational delivery mechanisms, carrier technologies, receptor-targeting strategies, or clinically established concepts.

Particular attention was given to the strength of evidence supporting targeting claims. Studies were critically assessed according to whether hepatic or cell-specific delivery was supported by appropriate non-targeted controls, quantitative biodistribution data, cell-resolved localization, receptor-dependent uptake, intracellular cargo release, or measurable pharmacodynamic effects. Whole-organ hepatic accumulation alone was not considered sufficient evidence of cell-specific targeting when the intended therapeutic target was a defined hepatic cell population.

For comparative evaluation, the selected studies were assessed according to the following criteria: delivery efficiency and biodistribution; distinction between organ-level accumulation and cell-specific uptake; cargo type, loading, stability, release, and, where relevant, endosomal escape; administration route; target-cell specificity and potential off-target uptake; safety and immunogenicity; physicochemical and analytical characterization; reproducibility and manufacturing scalability; and translational maturity. For biological and biomimetic platforms, additional consideration was given to source variability, identity, purity, potency, batch consistency, and storage stability.

Evidence was categorized according to the highest level directly supported for the individual system or product as in vitro, animal/preclinical, clinical-stage, approved, clinically used, or orphan-drug designated, where applicable. Regulatory designation was not considered equivalent to marketing approval. For clinical and regulatory information, official regulatory-agency records and ClinicalTrials.gov were preferentially used. Clinical-trial and regulatory information was last reverified on 1 September 2026. These criteria were applied throughout the review to critically compare synthetic, biological, and biomimetic delivery platforms and to distinguish experimental evidence of hepatic accumulation from evidence of cell-specific and functionally productive drug delivery.

## 3. Biological Basis and Disease-Dependent Determinants of Liver-Targeted Delivery

### 3.1. Hepatic Microanatomy and Determinants of Organ-Level Accumulation

The liver is anatomically and physiologically predisposed to interact with circulating drug carriers because of its high blood flow, specialized sinusoidal microvasculature, and prominent mononuclear phagocyte system [5,7,8,9,10]. The discontinuous sinusoidal endothelium facilitates exchange between the vascular compartment and the space of Disse, whereas LSECs and Kupffer cells exhibit high endocytic and phagocytic activity. These characteristics contribute to preferential hepatic accumulation of many systemically administered nanoscale delivery systems.

Hepatic accumulation at the whole-organ level, however, should not be considered synonymous with therapeutically relevant targeting. A substantial fraction of circulating nanoparticles may be sequestered by Kupffer cells and other components of the mononuclear phagocyte system, and high total liver-associated signal can therefore coexist with limited delivery to hepatocytes, HSCs, or other intended cellular targets [9,10]. This distinction is particularly important in imaging-based biodistribution studies, in which whole-organ fluorescence or radioactivity alone cannot identify the cellular compartment responsible for the signal.

The intrahepatic fate of a delivery system is determined by multiple interacting physicochemical characteristics, including particle size, morphology, surface charge, hydrophobicity, elasticity, and surface chemistry [5,8,9,10]. Rather than defining a universal “optimal” particle-size range for liver targeting, these variables should be interpreted in relation to carrier composition, route of administration, intended target cell, and disease state. Particle properties influence macrophage recognition, endothelial interactions, passage through the sinusoidal microenvironment, and access to parenchymal and non-parenchymal cell populations.

Thus, passive hepatic accumulation represents only the first stage of a multistep delivery process. For many therapeutic applications, successful delivery also requires escape from nonspecific phagocytic sequestration, access to the appropriate cellular compartment, internalization by the desired cell type, and release of biologically active cargo at an intracellular site where it can exert a measurable pharmacodynamic effect.

### 3.2. Disease-Associated Barriers and Altered Pharmacokinetics

The biological environment encountered by a delivery system differs substantially between healthy and diseased liver. Chronic hepatic disease can alter drug-metabolizing enzymes, membrane transporters, hepatic blood flow, plasma protein concentrations, biliary excretion, and other determinants of pharmacokinetics, leading to changes in drug exposure and drug–drug interaction risk [11]. These effects are relevant not only to free drugs but also to carrier systems because disease-associated changes in plasma composition, hepatic clearance, and cellular phenotype can modify carrier disposition.

Fibrosis creates particularly important physical and cellular barriers. Progressive deposition and cross-linking of extracellular matrix increase interstitial density and can restrict diffusion of nanocarriers toward HSCs and hepatocytes. In parallel, sinusoidal capillarization is characterized by loss of endothelial fenestrae and development of a more continuous basement-membrane-like structure, reducing access from the vascular lumen to the space of Disse [12,13,14]. Experimental studies have demonstrated that overcoming both capillarized endothelium and dense extracellular matrix can substantially improve delivery to activated HSCs [13], while restoration of sinusoidal fenestration has been shown to enhance access of targeted nanomedicines to fibrotic tissue [14].

Inflammatory liver injury introduces a different set of delivery determinants. Increased activity of hepatic macrophages and other components of the mononuclear phagocyte system may enhance sequestration and clearance of circulating nanocarriers, thereby altering their intrahepatic distribution [9,10]. In metabolic and advanced liver disease, changes in hepatic enzymes, transporters, cellular phenotype, and clearance mechanisms may alter pharmacokinetics and carrier disposition [11]. In hepatocellular carcinoma, vascular and cellular heterogeneity, fibrosis, and variable expression of molecular targets may further complicate extrapolation of targeting performance from healthy-liver or simplified tumor models [8].

These observations challenge the assumption that biodistribution measured in healthy animals accurately predicts behavior in diseased human liver. Disease stage should therefore be treated as a fundamental design variable rather than as a secondary experimental condition. The major disease-dependent barriers affecting hepatic drug delivery and their implications for carrier design are summarized in Table 1.

### 3.3. Molecular and Cellular Targets in the Liver

The therapeutic value of liver targeting depends on which cell population contributes most directly to the pathology being treated. Hepatocytes account for most liver mass and are central to metabolic disorders, viral infection, many inherited hepatic diseases, and production of circulating proteins. The asialoglycoprotein receptor (ASGPR), which recognizes galactose- and N-acetylgalactosamine-containing ligands, is among the best-characterized receptors for hepatocyte-directed delivery [15].

HSCs are major fibrogenic effector cells. During chronic injury, quiescent HSCs undergo activation and acquire a myofibroblast-like phenotype associated with extracellular matrix synthesis and altered expression of receptors such as PDGFRβ, CD44, CXCR4, and other surface molecules that have been exploited for targeted delivery [16]. Importantly, receptor abundance can change with disease stage, and a ligand validated in one model should not automatically be assumed to retain equal specificity across etiologies or stages of human fibrosis.

LSECs regulate exchange between blood and parenchyma, maintain sinusoidal homeostasis, and undergo major phenotypic changes during chronic injury [17]. Their high endocytic capacity makes them attractive targets but also means that apparent carrier uptake must be distinguished from delivery to hepatocytes located beyond the endothelial barrier.

Kupffer cells and recruited macrophages play important roles in inflammation, fibrogenesis, resolution, and nanoparticle clearance. Mannose-receptor-directed systems have been used experimentally to deliver siRNA to liver macrophage populations [18]. Neutrophils are relevant in acute inflammatory injury and can also be targeted through defined surface markers; CD177-mediated nanoparticle targeting has been demonstrated in human and murine neutrophils [19].

The existence of multiple potentially targetable populations means that “liver targeting” should be reported with sufficient cellular resolution. A carrier intended to modulate HSCs cannot be considered HSC-targeted solely because its whole-liver concentration is high, and a system intended for hepatocytes should be evaluated for competing uptake by Kupffer cells and LSECs.

### 3.4. Endogenous Targeting and the Protein Corona

After systemic administration, synthetic nanoparticles rapidly interact with proteins, lipids, and other biomolecules, creating a dynamic corona that determines their biological rather than merely synthetic identity. Corona composition can alter circulation time, cellular recognition, receptor engagement, and organ distribution [20].

The protein corona can enhance, redirect, or mask intended targeting. Adsorbed endogenous proteins may promote receptor-mediated uptake, while other corona components may sterically shield engineered surface ligands. Consequently, active targeting should not be conceptualized as a simple interaction between an exposed synthetic ligand and a receptor in an otherwise unchanged biological environment.

The translational implications are substantial because protein-corona composition differs between biological matrices and species. Human and mouse plasma can generate measurably different corona profiles on the same nanocarrier [21]. This provides one explanation for why targeting and biodistribution patterns observed in rodent models may not reproduce quantitatively in humans. Evaluation of targeted systems should therefore incorporate physiologically relevant biological fluids and, where possible, cross-species or human-derived models before clinical translation.

## 4. Carrier Platforms for Liver-Targeted Drug Delivery

### 4.1. Lipid-Based Delivery Systems

Lipid-based systems include conventional liposomes, solid lipid nanoparticles, nanostructured lipid carriers, lipid–polymer hybrids, and ionizable lipid nanoparticles (LNPs). Their principal advantages include high compatibility with nucleic-acid cargo, tunable composition, and established manufacturing technologies [6,22].

Formulation parameters strongly influence encapsulation, particle size, surface properties, stability, and biological activity. Microfluidic mixing has become an important manufacturing approach because it enables controlled and reproducible formation of LNPs containing macromolecular cargo [23]. However, formulation reproducibility does not by itself guarantee reproducible in vivo targeting: ionizable lipid structure, helper lipids, PEG-lipid content, particle composition, and interactions with endogenous proteins can substantially influence tissue and cell distribution.

For hepatocyte-directed RNA delivery, adsorption of endogenous proteins, particularly apolipoproteins, can contribute to hepatic uptake. Albumin-associated and lipid-composition-dependent effects have also been demonstrated in experimental mRNA delivery systems [24]. Other lipid compositions can shift delivery toward distinct cellular populations within the liver microenvironment [25]. These studies illustrate why “liver accumulation” and “hepatocyte delivery” must be distinguished experimentally.

A clinically established example of LNP-mediated hepatic RNA delivery is patisiran (Onpattro^®^), an intravenously administered siRNA LNP approved in 2018 for the treatment of polyneuropathy of hereditary transthyretin-mediated amyloidosis [26]. Patisiran reduces hepatic production of transthyretin and provides product-specific clinical evidence that productive RNA delivery to hepatocytes can be achieved. Importantly, its indication is a systemic disease driven by hepatic protein synthesis rather than a structural liver disease. Therefore, the clinical performance of patisiran should not be generalized to other LNP formulations or interpreted as evidence that LNPs as a platform provide equivalent delivery to hepatocytes, HSCs, macrophages, or malignant hepatic cells.

For RNA-loaded LNPs, productive delivery requires more than particle uptake. Internalization must be followed by endosomal escape and release of functional RNA into the appropriate intracellular compartment. Therefore, gene knockdown, protein expression, or another cargo-specific pharmacodynamic endpoint provides stronger evidence of functional delivery than fluorescent particle uptake alone.

Reported limitations and safety considerations for lipid-based nanoparticle systems include formulation-dependent inflammatory responses, complement interactions, PEG-related concerns, incomplete endosomal escape, and strong dependence of biodistribution on formulation composition [6,22]. The clinical maturity of this platform is best established for specific liver-directed nucleic-acid products rather than for LNPs as a class, as illustrated by patisiran [26].

### 4.2. Polymeric, Inorganic, and Protein-Based Nanoparticles

Polymeric nanoparticles comprise a heterogeneous group including PLGA particles, polymeric micelles, polymersomes, dendrimers, nanogels, and related systems. Their principal advantage is the ability to control degradation and cargo-release kinetics while enabling chemical conjugation of ligands or stimuli-responsive elements. Polymeric systems have been investigated extensively in preclinical models of fibrosis and cancer; however, their clinical translation remains limited by formulation complexity, residual solvent or reagent concerns, batch reproducibility, sterilization, scale-up, and uncertainties regarding long-term degradation products [27].

Inorganic nanoparticles include iron oxide, gold, silica, calcium phosphate, and other materials. These platforms can integrate imaging, magnetic manipulation, photothermal effects, or other physicochemical functions not readily achieved with conventional organic carriers [28]. Their safety and translational limitations are highly formulation- and material-dependent. Potential concerns include biodegradability, persistence, elemental toxicity, long-term tissue retention, and uncertainty regarding clinically acceptable clearance pathways [28].

Protein-based nanoparticles provide a different balance of properties. Albumin has been investigated as a carrier material because of its drug-binding capacity, biological origin, and potential interactions with disease-associated proteins [29]. Luo et al. developed albumin-based silibinin nanocrystals that showed enhanced uptake by activated HSCs and antifibrotic activity in experimental fibrosis [30]. This example demonstrates the potential of protein-based platforms, but it remains preclinical and should not be generalized to all albumin formulations. Source purity, structural integrity, protein modification, drug loading, aggregation, and reproducible characterization remain critical manufacturing considerations.

### 4.3. Nucleic Acid Nanostructures

DNA and other nucleic-acid nanostructures offer programmable size, geometry, sequence-defined functionalization, and the ability to integrate therapeutic and sensing functions [31]. DNA tetrahedra and related architectures can protect nucleic-acid cargo, present targeting ligands with defined spatial organization, and respond to environmental cues.

Programmable mechanocapsules and other responsive DNA structures illustrate the capacity of nucleic-acid nanotechnology to link molecular sensing with controlled cargo release [32]. In liver disease, Wei et al. reported a tetrahedral DNA nanoplatform with therapeutic activity in experimental acute liver failure [33].

Despite their structural precision, nucleic-acid nanostructures remain predominantly preclinical. Reported and anticipated translational barriers include nuclease stability, innate immune recognition, production cost, analytical complexity, pharmacokinetics, endosomal escape, and scale-up [31,32,33].

### 4.4. Mammalian-Derived Extracellular Vesicles

EV terminology requires particular care. According to MISEV2023 recommendations, extracellular vesicle is the preferred generic term for particles naturally released from cells that are delimited by a lipid bilayer, whereas the term exosome should be used only when an endosomal biogenesis pathway has been sufficiently demonstrated [34]. Accordingly, this review uses EV as the general term and retains “exosome” primarily when describing terminology used by the original authors or a specifically characterized biogenesis pathway.

EVs can carry proteins, lipids, nucleic acids, metabolites, and engineered therapeutic cargo. Their biological origin may provide cellular interaction properties that differ from those of fully synthetic carriers, while surface engineering can introduce additional targeting ligands. For example, engineered EV systems have been investigated for microRNA delivery to HSCs in experimental fibrosis [35].

Nevertheless, the properties of EVs should not be generalized as intrinsically superior to synthetic nanocarriers. Biocompatibility, immunogenicity, stability, tissue distribution, and targeting depend on source cell, culture conditions, isolation method, purification, dose, cargo, route of administration, and disease state. Systemically administered EVs may undergo substantial uptake by macrophages and other cells of the mononuclear phagocyte system rather than by the intended parenchymal population.

Clinical development also requires control of identity, purity, potency, cargo content, particle-to-protein ratio, process-related impurities, sterility, storage stability, and batch-to-batch consistency [36]. Isolation by ultracentrifugation, precipitation, size-exclusion chromatography, filtration, or chromatographic methods can yield preparations with different compositions and biological effects. Therefore, biological activity attributed to EVs should be linked to appropriately characterized preparations rather than to particle number alone.

For imaging studies, lipophilic dyes or persistent labels may dissociate from EVs and create misleading biodistribution signals. Direct hepatic delivery should ideally be confirmed using complementary methods, cell-resolved localization, functional cargo readouts, and appropriate free-label controls.

### 4.5. Plant-Derived Nanovesicles

Plant-derived nanovesicles (PDNVs) have attracted increasing attention because edible plant material can provide a potentially scalable source of lipid-rich nanoscale particles [37]. The term plant-derived nanovesicle is used here rather than “plant-derived exosome” or “exosome-like nanoparticle,” because mammalian exosome biogenesis should not be assumed for heterogeneous plant-derived vesicular preparations.

An important conceptual distinction is whether a plant vesicle is being studied as a natural therapeutic or as an engineered delivery carrier. Natural vesicles contain plant lipids, proteins, metabolites, and nucleic acids that may themselves produce biological effects. In contrast, an engineered carrier is evaluated primarily by its ability to encapsulate, protect, transport, and release an externally introduced therapeutic cargo. These functions should not be conflated.

Garlic-derived vesicles have demonstrated anti-inflammatory and hepatoprotective activity in experimental acute liver failure [38], whereas hemp sprout-derived vesicles have shown antifibrotic activity in animal models [39]. Pomegranate-derived vesicles containing endogenous ellagic acid have been associated with improvement of gut barrier dysfunction and liver injury in experimental MASLD [40]. Honeysuckle-derived vesicles have likewise demonstrated protective effects in acute liver failure, with evidence implicating modulation of the gut microbiota [41].

These oral studies highlight another important distinction: improvement of liver disease after oral administration does not necessarily prove direct vesicle transport from the gastrointestinal tract to hepatocytes or HSCs. Biological effects may arise through the intestinal barrier, microbiome, mucosal immune system, circulating metabolites, or other components of the gut–liver axis. Direct hepatic localization therefore requires experimental confirmation independent of therapeutic efficacy.

Engineered garlic-derived vesicles have also been investigated as oral carriers for astaxanthin in experimental alcohol-associated liver disease [42], illustrating the potential transition from naturally active particles to engineered drug-delivery systems.

Key translational considerations for PDNVs include variability related to plant species, cultivar, growth conditions, harvesting, extraction, and purification; incomplete definition of identity and potency; batch consistency; gastrointestinal stability; and the limited maturity of platform-specific regulatory standards [37]. Their potential scalability requires further demonstration under controlled pharmaceutical manufacturing conditions.

### 4.6. Cell-Mediated Delivery Systems

Cell-mediated delivery uses intact living or biologically active cells as carriers and should be distinguished clearly from systems that use isolated cell membranes. Erythrocytes, monocytes/macrophages, neutrophils, and other cells have been investigated as transport vehicles.

Erythrocytes have been investigated as carriers because of their prolonged circulation and capacity for intracellular cargo loading [43,44]. Their surface expression of CD47 contributes to self-recognition and reduced phagocytic clearance [45]. However, loading procedures can alter membrane integrity, deformability, osmotic stability, and clearance kinetics. Autologous production can reduce some immunological concerns but introduces patient-specific manufacturing complexity.

Neutrophils and macrophages have been investigated as delivery vehicles in inflammatory disease because of their recruitment toward chemotactic signals [46]. This property can be exploited for delivery into inflammatory microenvironments that may be less accessible to passive carriers. However, living cells are dynamic systems whose phenotype can change during isolation, loading, storage, and reinfusion.

More broadly, cell-based drug delivery faces logistical barriers including source selection, donor variability, ex vivo manipulation, sterility, release testing, viability, potency assays, cryopreservation, and individualized manufacturing [47]. These limitations currently restrict most intact-cell delivery approaches to experimental development.

### 4.7. Cell Membrane-Coated Biomimetic Nanoparticles

Cell membrane-coated nanoparticles differ fundamentally from intact-cell carriers. In these systems, an engineered nanoparticle core is coated with membrane isolated from erythrocytes, platelets, macrophages, or other cell sources. The goal is to combine the tunable cargo-loading properties of synthetic nanoparticles with selected surface characteristics of natural cells.

Erythrocyte-mimicking nanovehicles have been investigated for reproducing selected features of erythrocyte membranes, including prolonged circulation and membrane-mediated biological interactions, without using intact cells [48]. Platelet membrane coatings can provide interactions associated with vascular injury and inflammation [49], whereas macrophage membrane-coated systems are being explored for inflammatory homing [50].

An especially relevant liver example was reported by Xia et al., who developed HSC membrane-coated nanoparticles carrying TRAIL and demonstrated preferential interactions with activated HSCs and attenuation of fibrosis in experimental models [51].

The term cell membrane-coated nanoparticle is preferred over overlapping categories such as “natural cell-inspired nanoparticles” when the actual structure consists of a synthetic core surrounded by isolated biological membrane. This distinction eliminates ambiguity between intact-cell carriers and membrane-camouflaged synthetic nanoparticles.

Membrane coating does not automatically guarantee immune evasion or disease-specific targeting. Membrane isolation can alter protein orientation or composition, and preparation may contain intracellular contaminants. Critical quality attributes therefore include source-cell identity, membrane protein composition, coating completeness, particle size, surface charge, core-to-membrane ratio, residual impurities, stability, and biological potency.

### 4.8. Peptide-, Prodrug-, and Antibody-Based Strategies

Cell-penetrating peptides can promote intracellular transport of small molecules, proteins, nucleic acids, and nanoparticle-associated cargo [52]. Their principal functional role is to enhance cellular internalization; however, increased uptake does not necessarily confer cell specificity unless combined with a disease-associated targeting sequence or an activatable design [52].

Liver-directed prodrug strategies provide an alternative to physical carrier systems. Hepatic activation can exploit metabolic pathways or enzymes to increase local generation of active drug while reducing systemic exposure [53]. Such strategies may avoid some manufacturing challenges associated with nanoparticles but remain dependent on preserved hepatic enzymatic activity, which can vary substantially in advanced liver disease.

Antibodies and antibody–drug conjugates provide molecularly defined targeting strategies and have been investigated extensively in oncology, including hepatocellular carcinoma [54]. In liver applications, their large molecular size, Fc-mediated interactions, tumor penetration, antigen heterogeneity, immunogenicity, and manufacturing cost must be considered. As with ligand-functionalized nanoparticles, receptor binding alone is insufficient; effective therapy requires adequate access to the target, internalization when necessary, and functional release of the payload. A critical comparison of the principal liver-targeted delivery platforms, including their strengths, limitations, administration routes, target populations, and translational maturity, is provided in Table 2.

## 5. Cell-Specific and Intracellular Targeting Strategies

### 5.1. Targeting Hepatic Stellate Cells

Activated HSCs are major therapeutic targets in fibrosis because they contribute directly to extracellular matrix deposition and fibrotic remodeling [16]. Several targeting strategies exploit changes in receptor expression or the characteristic retinoid biology of HSCs.

Vitamin A-based targeting deserves particular clarification. Although retinol is transported systemically in association with retinol-binding proteins and participates in broader hepatic physiology, vitamin A-directed delivery has been investigated for HSC targeting because these cells represent the principal hepatic storage site for retinoids. A foundational study by Sato et al. used vitamin A-coupled liposomes carrying siRNA against the collagen-specific chaperone gp46 and demonstrated receptor-dependent delivery to HSCs and regression of experimental liver fibrosis [55]. Thus, vitamin A-based approaches should not be presented exclusively as hepatocyte-directed systems.

PDGFRβ has also been investigated as an HSC-associated target. Anti-PDGFRβ-coated gold nanorods preferentially accumulated in activated HSCs and produced antifibrotic effects following photothermal treatment in mice [56]. Cyclic pPB peptide-modified lipid nanoparticles have also been used to deliver siRNA to PDGFRβ-positive HSCs [57].

CD44-targeted approaches include hyaluronic acid-based nanomedicines. Antioxidative hyaluronic acid–bilirubin nanoparticles were reported to show enhanced uptake by activated HSCs together with antifibrotic activity in vivo [58]. EV-based systems carrying forsythiaside A have also been reported to exploit CD44-dependent uptake and modulate NLRP3-associated pyroptosis [59].

FGFR1-directed FGF2-conjugated superparamagnetic iron oxide nanoparticles reduced HSC activation in vitro and improved experimental acute liver injury in vivo [60].

Despite these encouraging results, receptor expression is not uniform across fibrosis etiologies or stages. Targeting claims should therefore include comparison with non-targeted carriers, evidence of receptor dependence, cell-resolved localization, and pharmacodynamic effects in the targeted HSC population.

### 5.2. Targeting Hepatocytes

Hepatocytes are the hepatic cell population with the strongest clinical validation to date for targeted RNA therapeutics. ASGPR is highly expressed on hepatocytes and binds terminal galactose and GalNAc residues, making it particularly suitable for ligand-directed internalization [15]. GalNAc conjugation has consequently been widely used for hepatocyte-directed oligonucleotide delivery [15].

Pullulan-based systems have also been explored for ASGPR-directed delivery. A multifunctional pullulan-stabilized iron oxide nanoprobe was evaluated for imaging and treatment of experimental liver fibrosis [61].

Patisiran provides a clinically established, product-specific example of hepatocyte-directed RNA delivery through an LNP formulation [26]. However, productive hepatocyte delivery should be demonstrated by gene silencing or protein-expression endpoints rather than by particle uptake alone.

Disease-associated changes must also be considered. Receptor expression, endocytic capacity, membrane composition, and intracellular trafficking can change during inflammation, malignancy, and advanced hepatic dysfunction. Thus, uptake data generated in healthy hepatocytes cannot automatically be extrapolated to cirrhotic or malignant tissue.

### 5.3. Targeting Kupffer Cells and Recruited Macrophages

For some therapeutic objectives, macrophage uptake is an obstacle; for others, it is the intended mechanism. Kupffer cells naturally sequester a substantial proportion of circulating nanomaterials [9,10]. This can reduce delivery to hepatocytes and HSCs but can be exploited for anti-inflammatory or macrophage-reprogramming therapies.

Phosphatidylserine-decorated nanoparticles have been used to exploit macrophage recognition pathways and enhance curcumin efficacy in experimental hepatic fibrosis [62]. Mannosyl-functionalized nanohydrogels have enabled siRNA delivery to immunosuppressive liver macrophages in vivo [18].

The major analytical challenge is therefore not simply to reduce macrophage uptake, but to determine whether it is therapeutically desirable for the intended indication. For a hepatocyte-targeted gene therapy, high Kupffer-cell uptake represents off-target sequestration; for a macrophage-targeted inflammatory therapy, the same biodistribution may be advantageous.

### 5.4. Targeting Liver Sinusoidal Endothelial Cells

LSECs are directly exposed to circulating carriers and possess strong scavenging and endocytic capacity. They are also functionally involved in inflammation, fibrosis, angiogenesis, and regulation of hepatic microcirculation [17].

Yu et al. developed a simvastatin-loaded nanoparticle system reported to preferentially interact with LSECs and remodel the tumor microenvironment in an experimental HCC model [63]. Such studies support LSECs as potential therapeutic targets but also highlight the need to distinguish endothelial uptake from transendothelial delivery to hepatocytes or HSCs.

Because LSECs undergo capillarization and other phenotype changes during chronic liver injury, targeting strategies should be evaluated in disease-relevant rather than exclusively healthy models.

### 5.5. Targeting Neutrophils and Other Immune Cells

Neutrophils rapidly migrate toward inflammatory signals and therefore represent both pathological mediators and potential delivery vehicles in acute liver injury. CD177-mediated nanoparticle targeting of human and murine neutrophils has been experimentally demonstrated [19].

Other leukocytes, including monocytes, macrophages, dendritic cells, and lymphocytes, may serve as targets or carriers depending on the indication. Cell-based and leukocyte-inspired systems exploit inflammatory homing but must be evaluated for unintended immune activation and changes in cell phenotype during ex vivo manipulation [47].

### 5.6. Evidence Required to Demonstrate Specific and Productive Delivery

A central methodological problem in targeted drug-delivery research is the tendency to equate increased tissue fluorescence with successful targeting. Evidence should instead be considered hierarchically.

At the first level, organ biodistribution establishes whether a carrier reaches the liver. At the second level, cell-resolved localization identifies the hepatic population responsible for uptake. At the third level, receptor-dependent specificity is supported by non-targeted carrier controls, competition or receptor-blocking experiments, receptor-deficient models, or other mechanistic evidence. At the fourth and most relevant level, productive intracellular delivery requires demonstration that cargo reaches the functional intracellular compartment and produces the intended molecular or pharmacological effect.

For RNA therapeutics, productive delivery should be assessed through gene knockdown, editing, or protein expression rather than total particle-associated signal. Endosomal escape is often a major bottleneck. For EVs, functional delivery should be distinguished from uptake of membrane components or label. For orally administered PDNVs, direct liver transport should be distinguished from effects mediated through the gastrointestinal tract and gut–liver axis.

Cross-species translation presents another challenge. LNP performance and cellular responses can vary markedly between murine, non-human primate, and human hepatocyte models [64]. This reinforces the need for human-relevant systems before assuming that targeting efficiency measured in rodents will translate quantitatively to patients. Representative cell-specific targeting strategies, the evidence required to demonstrate specificity, and their major translational barriers are summarized in Table 3.

## 6. Clinical Translation, Manufacturing, Analytical Characterization, and Regulatory Considerations

### 6.1. Approved Liver-Directed Systems and Clinical Pipeline

The most clinically mature examples of liver-directed delivery are specific RNA therapeutics that exploit hepatocyte biology. Importantly, these approved products provide product-specific evidence of therapeutically effective hepatic delivery even when the treated disease manifests substantially outside the liver.

Patisiran is an intravenously administered siRNA LNP approved in 2018 for polyneuropathy of hereditary transthyretin-mediated amyloidosis [26]. Its therapeutic activity depends on productive siRNA delivery to hepatocytes and subsequent reduction in hepatic transthyretin synthesis.

Givosiran (Givlaari^®^) is a GalNAc-conjugated siRNA approved for acute hepatic porphyria. The GalNAc ligand enables hepatocyte-directed uptake after subcutaneous administration, and FDA marketing approval was granted in 2019 [65]. Lumasiran (Oxlumo^®^) is another GalNAc-conjugated siRNA, approved in 2020 for primary hyperoxaluria type 1; it reduces hepatic oxalate production by silencing HAO1 [66]. Together, givosiran and lumasiran provide product-specific clinical evidence that GalNAc-mediated hepatocyte delivery can support therapeutically effective RNA interference.

The clinical landscape for treatment of structural liver disease is less mature. Fazirsiran, an RNA-interference therapeutic directed against mutant alpha-1 antitrypsin production in hepatocytes, produced substantial reductions in intrahepatic Z-AAT in a phase 2 study [67]. The ongoing Phase 3 study NCT05677971 in alpha-1 antitrypsin deficiency-associated liver disease with fibrosis was listed as Recruiting on ClinicalTrials.gov when last verified on 1 September 2026 [68].

Preclinical studies are also exploring greater cell-level control of hepatic delivery. A 2026 study described an LNP formulation that showed hepatocyte-preferential mRNA delivery together with therapeutic effects in an experimental liver-fibrosis model [69]. This finding is specific to the reported formulation and preclinical model and should not be generalized to LNPs as a platform.

EV therapeutics remain at an earlier stage. NCT07409259, registered under the title “PT-MSCs Exosome Injection in the Treatment of Chronic-to-acute Liver Failure,” is an Early Phase 1 study in patients with acute-on-chronic liver failure and was listed as Active, not recruiting when last verified on 1 September 2026 [70]. The FDA Orphan Drug Designations and Approvals database lists Signal regulatory protein alpha (SIRPalpha)-Exosome as designated on 25 January 2024 for treatment of acute liver failure regardless of etiology; the same FDA record states that the product is not FDA approved for the orphan indication [71]. Representative clinically approved, clinical-stage, and selected preclinical liver-directed delivery systems are summarized in Table 4.

The translational landscape therefore remains highly asymmetric. Clinical validation is strongest for specific hepatocyte-directed RNA products, whereas the HSC-, LSEC-, macrophage-, biomimetic membrane-, PDNV-, and most EV-based systems discussed in this review remain predominantly at the preclinical stage.

### 6.2. Manufacturing and Analytical Characterization

Translation of a promising carrier requires a reproducible pharmaceutical product rather than only a biologically active laboratory preparation. Critical quality attributes differ across platform classes but commonly include particle size and distribution, morphology, surface charge, composition, encapsulation efficiency, free versus encapsulated cargo, release kinetics, stability, sterility, endotoxin content, and biological potency.

For liposome drug products, FDA guidance emphasizes robust chemistry, manufacturing, and controls (CMC), together with appropriate pharmacokinetic and bioavailability characterization [72]. More broadly, scale-up of lipid-based nanoparticle manufacturing can alter mixing conditions, particle composition, and product attributes, making process control and comparability assessment important during development.

EV development presents additional analytical complexity because product identity depends on both biological source and isolation process. Therapeutic EV preparations require characterization of particle and protein content, purity, potency, source-cell properties, contaminants, batch consistency, storage conditions, and stability [36,73]. A single marker or nanoparticle tracking profile is insufficient to establish pharmaceutical identity.

Cell membrane-coated nanoparticles introduce a hybrid quality-control problem. Both the synthetic core and the biological membrane require characterization, while membrane coating must be demonstrated rather than assumed. Intact-cell carriers require additional testing of cell identity, viability, function, sterility, phenotype, and potency [43,44,45,46,47].

PDNVs face analogous challenges related to raw-material variability, extraction, purification, and product definition. Pharmaceutical development will require specifications for source plants, cultivation or procurement, extraction and purification processes, cargo composition, particle characteristics, microbiological quality, stability, and biological potency [37].

### 6.3. Safety, Immunogenicity, and Regulatory Challenges

Safety is product-, formulation-, and context-dependent. Synthetic nanoparticle formulations may activate complement or innate immune pathways, undergo mononuclear-phagocyte-system sequestration, or produce formulation-specific toxicities. Inorganic materials may raise material-dependent concerns regarding persistence and long-term clearance. Biological platforms introduce different risks related to source material, contaminants, immunogenic components, transmissible agents, and variability in biological potency.

For EV-based products, regulatory maturity remains limited [73]. Recent FDA enforcement activity in 2026 continued to address products marketed as exosome therapies that were considered unapproved new drugs and/or unlicensed biological products, underscoring the need to distinguish experimental or marketed use from regulatory approval [74].

Another translational problem is the widespread use of generalized terms such as “biocompatible,” “non-immunogenic,” or “naturally targeted.” Such characteristics must be demonstrated for a specific product, source, dose, manufacturing process, and route of administration. A favorable outcome in one animal model does not establish platform-wide safety.

Finally, animal-to-human translation remains a fundamental limitation. Differences in immune systems, plasma protein composition, receptor expression, organ size, disease stage, and cellular uptake can substantially alter biodistribution [21,64]. Robust clinical translation therefore requires a progression from mechanistic cell studies to disease-relevant animal models, human-derived models, scalable manufacturing, validated analytical assays, and carefully designed early-phase clinical studies.

## 7. Conclusions

Liver-targeted drug delivery research has increasingly moved beyond a focus on preferential organ accumulation toward a more demanding paradigm of disease-adapted, cell-resolved, and functionally productive delivery. The liver is naturally accessible to circulating nanomaterials, but this accessibility creates both an opportunity and a challenge: high whole-organ accumulation frequently reflects clearance by Kupffer cells and does not necessarily indicate therapeutic delivery to hepatocytes, HSCs, LSECs, or malignant cells.

Synthetic platforms provide substantial opportunities for engineering control over composition, cargo loading, and surface properties. Among the systems reviewed here, clinical validation is strongest for specific hepatocyte-directed RNA products, including patisiran and approved GalNAc-siRNA therapeutics. These products provide clinical evidence that productive hepatic gene silencing can be achieved in defined therapeutic settings. By contrast, the polymeric, inorganic, HSC-directed, LSEC-directed, and biomimetic nanoparticle systems discussed in this review remain predominantly at the preclinical stage.

Biological delivery platforms offer complementary design opportunities but also introduce distinct sources of variability and uncertainty. EV properties depend strongly on cellular source, production, purification, characterization, dose, cargo, and route of administration. Their potential should therefore be evaluated without assuming universal biocompatibility or intrinsic targeting. Similarly, PDNVs may function as natural therapeutics, engineered carriers, or both, and oral hepatoprotective effects should not automatically be interpreted as evidence of direct vesicle delivery to liver cells.

Cell-mediated and cell membrane-coated systems should also be treated as distinct categories. Intact cells retain active biological functions and migratory behavior but require complex cell manufacturing, whereas membrane-coated nanoparticles combine a synthetic core with selected membrane-associated properties and introduce a different set of analytical and scale-up requirements.

Disease state is an important determinant of delivery performance across platform classes. Fibrotic matrix deposition, sinusoidal capillarization, inflammation, altered drug metabolism, receptor heterogeneity, and changes in protein corona formation can substantially modify delivery. For this reason, targeting systems should be validated in pathophysiologically relevant models rather than extrapolated from healthy liver.

Future development should focus less on maximizing total liver accumulation and more on demonstrating four sequential requirements: delivery to the liver, localization within the intended cell population, productive intracellular release, and a measurable therapeutic effect. Advancement to clinical use will additionally require reproducible manufacturing, rigorous analytical characterization, realistic safety assessment, and evidence that targeting mechanisms remain functional in human disease.

Future advances are unlikely to be defined by a single carrier class. Progress will instead depend on integrating synthetic engineering, biological interfaces, disease-adapted targeting strategies, reproducible manufacturing, and rigorous analytical characterization while maintaining clear evidence standards at each stage of development.

## Figures and Tables

**Table 1:** Disease-dependent barriers relevant to liver-targeted delivery.

Pathological Context	Major Delivery Barriers	Potential Consequences for Carriers	Design Implications	References
Fibrosis/cirrhosis	Sinusoidal capillarization, loss of fenestrae, dense and cross-linked extracellular matrix, and altered HSC phenotype	Reduced penetration into the space of Disse; restricted diffusion; heterogeneous carrier distribution; altered accessibility of cellular targets	Disease-adapted carrier properties; strategies facilitating sinusoidal or extracellular-matrix penetration; validation of targeting approaches in fibrosis-relevant models	[12,13,14]
Inflammatory liver injury	Enhanced macrophage and mononuclear phagocyte-system activity	Increased phagocytic sequestration and clearance; reduced availability of circulating carriers for parenchymal target cells	Macrophage uptake should be considered according to the therapeutic objective: it may be advantageous for macrophage-directed therapy but may represent off-target sequestration for other hepatic targets	[9,10]
Metabolic liver disease	Altered hepatic enzymes, membrane transporters, cellular phenotype, and clearance mechanisms	Changes in pharmacokinetics, hepatic exposure, cellular uptake, and carrier disposition	Disease-specific evaluation of dose, biodistribution, target accessibility, and pharmacodynamic activity	[11]
Hepatocellular carcinoma	Vascular and cellular heterogeneity, fibrosis, and variable expression of molecular targets	Heterogeneous tumor access and potential off-target uptake by non-malignant hepatic cells	Targeting claims should be supported by tumor- and cell-resolved localization together with functional antitumor readouts	[8]
Advanced hepatic dysfunction	Reduced metabolic capacity, altered protein binding, transporter activity, and biliary clearance	Altered or unpredictable systemic exposure and carrier disposition	Reassessment of dose, pharmacokinetics, clearance, and drug–drug interaction potential	[11]

Abbreviations: HSC, hepatic stellate cell.

**Table 2:** Critical comparison of major liver-targeted delivery platforms.

Platform	Principal Strengths	Major Limitations and Safety Concerns	Typical Administration Route	Principal Hepatic Targets/Applications	Translational Maturity	References
Lipid nanoparticles/liposomes	High nucleic-acid loading; tunable composition; established manufacturing approaches	Formulation-dependent immune and complement effects; PEG-related concerns; incomplete endosomal escape; formulation-dependent biodistribution	Mainly IV; SC for selected ligand-conjugated RNA products	Primarily hepatocytes for established RNA delivery; experimental delivery to other hepatic cell populations	Approved/clinically used for selected liver-directed RNA products; otherwise formulation-dependent	[6,22,23,24,25,26]
Polymeric nanoparticles	Controlled release; diverse chemistry; ligand functionalization; stimuli-responsive design	Complex synthesis; residual reagents; degradation products; reproducibility and scale-up challenges	Mainly IV	HSCs, macrophages, and hepatic tumors depending on formulation	Animal/preclinical	[27]
Inorganic nanoparticles	Imaging, magnetic, photothermal, and theranostic functions	Material-dependent persistence, toxicity, tissue retention, and clearance concerns	Mainly IV or local	Fibrotic liver and hepatic tumors in experimental models	Animal/preclinical	[28]
Protein-based nanoparticles	Drug-binding capacity; biological carrier materials; potential receptor-associated interactions	Aggregation; source purity; formulation-dependent loading; stability and analytical characterization	Mainly IV	Activated HSCs and fibrotic liver in representative experimental systems	Animal/preclinical	[29,30]
Nucleic-acid nanostructures	Programmable geometry and functionalization; integration of sensing and therapeutic functions	Nuclease degradation; innate immune recognition; production cost; analytical complexity; endosomal escape	Mainly IV in experimental models	Acute liver injury and other experimental liver applications	Animal/preclinical	[31,32,33]
Mammalian extracellular vesicles	Biological interfaces; capacity to carry endogenous or engineered cargo	Heterogeneity; macrophage sequestration; variable yield; identity, purity, potency, loading, storage, and batch-consistency challenges	IV, local, or other experimental routes	HSCs, hepatocytes, and macrophages depending on source and engineering	Predominantly animal/preclinical; selected clinical-stage investigations	[34,35,36]
Plant-derived nanovesicles	Potential oral administration; potentially scalable biological source; intrinsic bioactive cargo in some preparations	Source variability; uncertain identity and gastrointestinal fate; batch consistency; limited platform-specific regulatory standards	Primarily oral in current liver studies	Liver injury, fibrosis, MASLD, and alcohol-associated liver disease in experimental models	Animal/preclinical	[37,38,39,40,41,42]
Intact cell-mediated systems	Natural circulation or inflammatory homing; capacity to carry diverse cargo	Cell viability; phenotype alteration; donor variability; sterility; individualized and complex manufacturing	Mainly IV	Inflammatory and macrophage-rich lesions; systemic delivery applications	Predominantly animal/preclinical	[43,44,45,46,47]
Cell membrane-coated nanoparticles	Synthetic cargo-loading core combined with selected biological membrane properties	Membrane-source variability; incomplete coating; altered protein orientation; contamination; analytical and scale-up challenges	Mainly IV	Fibrosis, inflammation, and cancer depending on membrane source	Animal/preclinical	[48,49,50,51]
Peptide-assisted systems	Enhanced cellular internalization; modular functionalization	Limited cell specificity of some CPPs; proteolysis; possible off-target uptake	Platform-dependent	Intracellular delivery to selected hepatic cell populations	Predominantly animal/preclinical	[52]
Liver-directed prodrugs	No particulate carrier required; hepatic metabolic activation can increase local drug generation	Dependence on hepatic enzyme activity; disease-related metabolic variability	Oral or systemic, depending on molecule	Primarily hepatocyte-associated metabolic activation	Product-dependent	[53]
Antibody/antibody–drug systems	Molecularly defined target recognition; modular payload design	Antigen heterogeneity; Fc-mediated effects; limited tissue penetration; immunogenicity; conjugation and manufacturing complexity	Mainly IV	HCC and molecularly defined hepatic targets depending on product	Product- and target-dependent; clinical maturity should be assigned to individual products rather than the platform class	[54]

Abbreviations: CPP, cell-penetrating peptide; EV, extracellular vesicle; HCC, hepatocellular carcinoma; HSC, hepatic stellate cell; IV, intravenous; MASLD, metabolic dysfunction-associated steatotic liver disease; PEG, polyethylene glycol; SC, subcutaneous.

**Table 3:** Cell-specific targeting strategies, evidence requirements, barriers, and development stage.

Target Cell Population	Representative Targets or Strategies	Evidence Required to Support Cell Specificity	Major Delivery Barriers and Limitations	Development Stage	References
Activated hepatic stellate cells	Vitamin A/retinoid-associated targeting; PDGFRβ; CD44; FGFR1	Non-targeted controls; HSC-resolved localization; receptor-dependent uptake; intracellular cargo release; antifibrotic pharmacodynamic response	Sinusoidal capillarization; dense fibrotic ECM; receptor heterogeneity; disease-stage dependence	Animal/preclinical	[55,56,57,58,59,60]
Hepatocytes	ASGPR/GalNAc; pullulan-based systems; LNP-mediated RNA delivery	Hepatocyte-resolved localization; receptor dependence where applicable; gene knockdown or protein-expression readouts for RNA cargo	Kupffer-cell and LSEC sequestration; endosomal escape; disease-dependent changes in receptor expression and intracellular trafficking	Approved/clinically used for selected RNA products; other represented systems animal/preclinical	[15,26,61]
Kupffer cells/hepatic macrophages	Mannose receptor; phosphatidylserine; exploitation of phagocytic uptake	Macrophage-resolved uptake; comparison with non-targeted systems; measurable immunological or pharmacodynamic effect	Macrophage heterogeneity; nonspecific MPS uptake; phenotype-dependent responses	Animal/preclinical	[9,10,18,62]
Liver sinusoidal endothelial cells	Endocytic/scavenger pathways; endothelial-directed nanoparticles	LSEC-resolved uptake; comparison with hepatocyte and Kupffer-cell uptake; endothelial pharmacodynamic response	Capillarization; phenotypic changes; difficulty distinguishing endothelial uptake from transendothelial delivery	Animal/preclinical	[17,63]
Neutrophils	CD177-mediated targeting; inflammatory homing	Receptor-dependent uptake; non-targeted controls; validation in relevant inflammatory models	Short lifespan; activation risk; species-dependent receptor biology	Preclinical	[19]
Monocytes and other leukocytes	Inflammatory homing; cell-mediated or biomimetic approaches	Cell-resolved trafficking; appropriate controls; functional immune or pharmacodynamic response	Phenotype alteration; off-target immunomodulation; effects of ex vivo manipulation	Preclinical	[46,47]

Abbreviations: ASGPR, asialoglycoprotein receptor; ECM, extracellular matrix; FGFR1, fibroblast growth factor receptor 1; GalNAc, N-acetylgalactosamine; HSC, hepatic stellate cell; LNP, lipid nanoparticle; LSEC, liver sinusoidal endothelial cell; MPS, mononuclear phagocyte system; PDGFRβ, platelet-derived growth factor receptor beta. Development stage refers to the highest evidence level supported by the representative systems cited in each row and should not be generalized to all targeting strategies directed toward the corresponding cell population.

**Table 4:** Representative translational and regulatory landscape of liver-directed delivery systems.

System	Platform/Cargo	Primary Hepatic Target	Indication	Evidence/Regulatory Status	Route	Key Translational Significance	References
Patisiran (Onpattro^®^)	siRNA lipid nanoparticle	Hepatocytes	Polyneuropathy of hereditary transthyretin-mediated amyloidosis	Approved/clinically used; FDA approval, 2018	IV	Product-specific clinical precedent for productive LNP-mediated siRNA delivery to hepatocytes	[26]
Givosiran (Givlaari^®^)	GalNAc–siRNA conjugate	Hepatocytes/ASGPR	Acute hepatic porphyria	Approved/clinically used; FDA approval, 2019	SC	FDA-approved GalNAc–siRNA example supporting receptor-mediated hepatocyte delivery	[65]
Lumasiran (Oxlumo^®^)	GalNAc–siRNA conjugate	Hepatocytes/ASGPR	Primary hyperoxaluria type 1	Approved/clinically used; FDA approval, 2020	SC	FDA-approved GalNAc–siRNA example demonstrating therapeutic modulation of a hepatic metabolic pathway	[66]
Fazirsiran	RNAi therapeutic	Hepatocytes	Alpha-1 antitrypsin deficiency-associated liver disease with fibrosis	Clinical-stage; Phase 2 clinical evidence; Phase 3 NCT05677971 recruiting	SC	Clinical-stage hepatocyte-directed RNAi approach being evaluated for progressive liver disease and fibrosis	[67,68]
Hepatocyte-preferential OSK mRNA LNP	mRNA lipid nanoparticle	Hepatocytes	Experimental liver fibrosis	Animal/preclinical	IV	Demonstrated hepatocyte-preferential mRNA delivery and therapeutic effects in the reported preclinical fibrosis model; findings are formulation-specific	[69]
PT-MSC-derived EV preparation	Extracellular-vesicle-based biological preparation	Hepatic target population not definitively established in humans	Acute-on-chronic liver failure	Clinical-stage; Early Phase 1; NCT07409259 Active, not recruiting	IV	Demonstrates entry of an EV-based therapeutic preparation into exploratory human investigation; cell-specific hepatic delivery remains unestablished	[70]
Signal regulatory protein alpha (SIRPalpha)-Exosome	EV-based product	Specific hepatic cellular target not established by the regulatory designation	Acute liver failure regardless of etiology	Orphan-drug designated by FDA, 25 January 2024; not FDA approved for the orphan indication	Not established from the FDA orphan-designation record	Illustrates regulatory designation of an EV-based candidate; orphan-drug designation does not establish marketing approval, clinical efficacy, or cell-specific hepatic targeting	[71]

Abbreviations: ASGPR, asialoglycoprotein receptor; EV, extracellular vesicle; FDA, U.S. Food and Drug Administration; GalNAc, N-acetylgalactosamine; IV, intravenous; LNP, lipid nanoparticle; mRNA, messenger RNA; OSK, OCT4/SOX2/KLF4; PT-MSC, placenta-derived mesenchymal stem cell; RNAi, RNA interference; SC, subcutaneous; siRNA, small interfering RNA. Clinical-trial and regulatory information current as of 1 September 2026.

## Data Availability

No new data were created or analyzed in this study. Data sharing is not applicable to this article.
